# Waiting for elective orthopaedic surgery: a qualitative scoping review of the patient experience

**DOI:** 10.1093/intqhc/mzag164

**Published:** 2026-10-10

**Authors:** Elizabeth Walkley, Katheryn Hall, Dannielle Hopkinson, Mina Nichols-Boyd, Elise Williams, Nicole Pope

**Affiliations:** Orthopaedic Surgery Unit, Austin Health, Heidelberg, Victoria, Australia; Department of Surgery, University of Melbourne, Austin Precinct, Victoria, Australia; Department of Surgery, University of Melbourne, Austin Precinct, Victoria, Australia; Patient Safety & Clinical Excellence Unit, Austin Health, Heidelberg, Victoria, Australia; Austin Health Sciences Library, Austin Health, Heidelberg, Victoria, Australia; Patient Safety & Clinical Excellence Unit, Austin Health, Heidelberg, Victoria, Australia; Monash Centre for Health Research and Implementation, Monash University, Melbourne, Victoria, Australia

**Keywords:** elective surgery, orthopaedic, planned surgery, patient experience, quality of life, waiting list

## Abstract

**Background:**

Public health waiting lists for elective or planned orthopaedic surgery are increasing to the point where demand far exceeds our ability to treat. This is largely due to ageing populations, rising obesity and the continued impact of delays caused by the COVID-19 pandemic. While waiting, patients suffer debilitating physical pain, functional limitation, social isolation, and associated mental health impacts while some are unable to work. This qualitative scoping review explored and mapped the experiences of patients of waiting for elective or planned orthopaedic surgery.

**Methods:**

This review was conducted according to the Joanna Briggs Institute Scoping Review methodology and reported in line with the Preferred Reporting Items for Systematic Reviews and Meta-Analyses extension for scoping reviews. A comprehensive database search was conducted to identify English-language articles from 2009 until June 2025. Data extraction was informed by the Standards for Reporting Qualitative Research. Results were thematically analysed and presented using the Quality Health Outcomes Model.

**Results:**

The initial search identified 4713 articles, of which 21 manuscripts met inclusion criteria. The 21 articles comprised 17 primary qualitative research studies and four review articles published within the designated time period (2009–2025). Two key themes were identified: (i) patients waiting for elective orthopaedic surgery need psychosocial support and (ii) system-level, multifactorial communication issues with the waiting list leave patients feeling lost between primary and specialist care. These themes suggest that the challenges faced due to lengthy elective surgery waiting lists are not simply a capacity issue, but also a challenge of coordinated, patient- centred care.

**Conclusion:**

Waiting for surgery is consistently described by patients as a source of anxiety and distress. With waiting list times for elective surgery increasing, the experience can be improved in part with adequate communication from healthcare providers. A re-focus on what matters most to the patient and their family is required in order to deliver adequate patient-centred care while patients wait for surgery.

Note ‘elective surgery’ can also be referred to as ‘planned surgery’ and these terms can be used interchangeably. For the purposes of this paper, only the term ‘elective’ will be used throughout.

## Introduction

Waiting in public healthcare is inevitable where demand exceeds supply, and elective surgery lists are no exception [1, 2]. Orthopaedic surgery is particularly impacted by waiting list delays. Ageing populations, obesity-related illness and the increasing prevalence of osteoarthritis continue to impact public patients needing elective orthopaedic surgery in high income countries, compounded by disproportionate surgery cancellations occurring during the COVID-19 pandemic [3–5]. These waiting times are regularly scrutinized and pose a significant concern for the general public [3, 6]. Ethical challenges inherently exist in the management of elective surgery waiting lists, where clinical need is directed by surgeons but psychosocial factors or deterioration in health-related quality of life over time are often overlooked [7, 8].

While increased surgical activity improves throughput and reduces average waiting times, patients currently on the list continue to suffer physical and psychological health deterioration including loss of independence and impaired mobility [9, 10]. These experiences are not equally distributed across population groups [11]. A post-COVID analysis of waiting lists in the National Health Service (NHS) found that patients living in impoverished regions are twice as likely to wait over a year longer for surgery than those living in more affluent areas, while those in lower socioeconomic locations often suffer an increase in co-morbidities and are further disadvantaged by increased financial strain, carer demands and family stress [9, 12].

Literature to date has demonstrated that longer waits for elective orthopaedic surgery are correlated with worse postoperative outcomes and delayed surgical care also contributes to the burden of chronic disease on the primary healthcare system [11, 13, 14]. While less research has focused on the psychological needs of patients waiting for surgery, a post-COVID literature review sought to understand the psychosocial impacts of delayed orthopaedic surgery, including financial consequences and changes in productivity and employment [15]. Research in this space is predominantly quantitative in nature, not specific to orthopaedics, and rarely focuses on the waiting period itself or the qualitative perspective required to assess the impact and needs of waiting patients.

### Scoping review objectives

Caring for patients on the waiting list for elective orthopaedic surgery requires an in-depth understanding of the experience of individuals waiting for surgery and a patient-centred response to the issues identified. This scoping review mapped and synthesized qualitative evidence to answer the question, ‘What is the experience of adults awaiting elective or planned orthopaedic surgery in the public healthcare system?’

## Materials and methods

### Protocol and registration

This scoping review was conducted according to the Joanna Briggs Institute (JBI) methodology and reported using the Preferred Reporting Items for Systematic Reviews and Meta-Analyses extension for scoping reviews (PRISMA-ScR) [16, 17]. The protocol was registered in Open Science Framework: https://doi.org/10.17605/OSF.IO/VZBMU.

### Search strategy and information sources

An iterative approach was used by a clinical librarian (MN-B) to develop the search strategy (run from 2009 to 6 June 2025). Through preliminary searches in Ovid MEDLINE and Google Scholar, relevant papers were selected that made up the gold set. Utilizing Yale MeSH Analyzer [18], the gold set was examined for relevant MeSH terms and keywords/phrases for the two main concepts: wait lists and orthopaedic surgery, and a qualitative search filter from CDA/AMC was applied [19]. The Ovid MEDLINE and Embase search strategies were peer-reviewed by another librarian using the PRESS checklist [20], before translation to other databases. The search strategies for each database can be found in the Supplementary Materials.

### Selection of sources of evidence (inclusion/exclusion criteria)

Inclusion criteria were qualitative studies and qualitative evidence syntheses of the adult patient experience of awaiting elective orthopaedic surgery in the public healthcare setting. Studies on cancer surgery, trauma, and emergency procedures were not considered ‘elective’ [1]. Literature sources included publications in peer-reviewed journals, grey literature and white papers. Review articles relating to public health patient experience of waiting for elective orthopaedic surgery were included if they reported on primary qualitative research in line with our published protocol. Reference lists were manually searched to identify additional sources, and duplications were removed. Earlier systematic reviews are now outdated [1] and did not specifically focus on orthopaedic surgery or the waiting period [21]. Three researchers (ETW, DH, EW) screened all titles, abstracts, and full text in duplicate. Any discrepancies were discussed with the supervising co-author (NP). Forward and backward citation searching was also conducted by the primary author for each included paper, yielding an additional 21, which were imported into Covidence and re-screened. All eligible articles were imported into Covidence (Veritas Health Innovation, Melbourne, Australia) for screening, and duplicates were removed. Consistent with standards for scoping reviews, we did not conduct any quality appraisals of the included papers [17].

### Data charting process

The data extraction template was developed using JBI and the Standards for Reporting Qualitative Research (SRQR) checklist [16, 22]. The initial draft was piloted by all four reviewers with one article and refined based on group consensus and shared understanding of key terms. All included articles were double-extracted in Covidence by the primary author (ETW) and one of the three co-authors (DH, EW, NP). Data extracted included study location, data collection methods (e.g. interviews), recruitment strategy, analytic techniques, and a summary of results.

### Synthesis of results

Study findings are grouped into themes and presented using the Quality Health Outcomes Model (QHOM). This model was chosen as it shows the bi-directional influence of patient experience on outcomes and interventions, where patient and system factors intersect the relationship between interventions and their outcomes in healthcare [23]. The four elements of the QHOM; client, intervention, outcome, and system were applied at the analysis phase in the context of the elective surgery patient journey. The intervention and outcome were iteratively defined through analysis of the study findings.

## Results

### Selection of sources of evidence

A total of 7166 articles were found using this search strategy, of which 2453 duplicates were removed. Title and abstracts of the remaining 4713 articles were screened, and 73 full-text studies were assessed for eligibility (see PRISMA-ScR diagram in Fig. 1). Final consensus revealed a total of 21 studies included.

**Figure 1:**
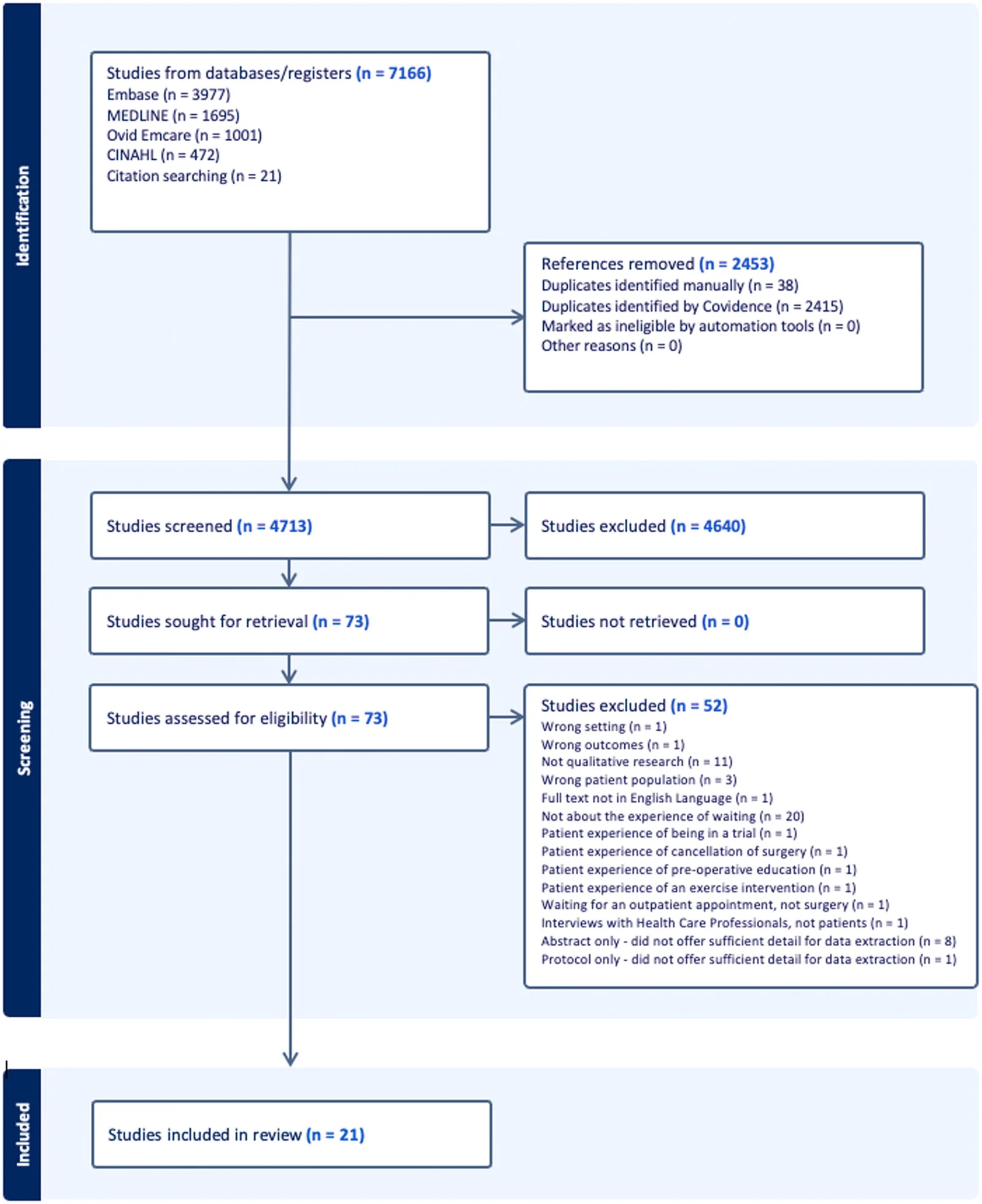
PRISMA-SCR flow diagram: public health patient experience of elective orthopaedic surgery waiting lists—a qualitative scoping review. 7166 studies were screened including an additional 21 manually added from citation searching, and 21 papers were included.

### Characteristics of sources of evidence

The 21 articles comprised 17 primary qualitative research studies and four review articles published within the designated time period. All studies were conducted in high-income countries. A summary of the review papers can be found in Table 1. Two papers, published three years apart in different journals, discussed results from the same primary qualitative study; however, both were included as they independently met the review criteria. Of the review articles, three considered both qualitative and quantitative literature sources, and for the purposes of this scoping review, only content from the qualitative studies was discussed. Each review paper discussed findings in relation to the study design; thus, data extraction for this review only included information relevant to the qualitative papers. None of the reviews were both qualitative and solely focussed on orthopaedic surgery. Two review articles which analysed a combined 14 qualitative studies included five of the 17 primary papers presented in this review [24–28]; however, exemplar quotes are only sourced from each paper once.

**Table 1:** Summary review characteristics.

Category	*n*	%
Article type		
Primary qualitative research [9, 24–28, 30–33, 35–41]	17	81
Review article [1, 15, 21, 51]	4	19
Location of study		
United Kingdom [9, 15, 26, 28, 35, 38–41]	9	43
Canada [1, 24, 25, 31, 32]	5	24
Italy [30, 33, 36]	3	14
Sweden [27, 37]	2	10
Australia [21]	1	5
Several [51]	1	5
Patient population		
Orthopaedic [9, 26–28, 30, 32, 33, 35–40, 51]	14	67
Orthopaedic and other [1, 15, 21, 24, 25, 31, 41]	7	33
Qualitative data collection methods^a^		
Interviews [9, 24–28, 30–33, 35–37, 39–41]	16	94
Focus group [38]	1	6
Sample size^a^		
<10 participants [9, 28, 37–39, 41]	6	35
10–20 participants [27, 30, 31, 36, 40]	5	30
>20 participants [24–26, 32, 33, 35]	6	35
Recruitment strategy^a^		
Via treating clinician [24, 25, 28, 32, 33, 35, 37]	7	41
Via local waiting list [26, 27, 30, 31, 36, 39]	6	35
Nested within a randomized controlled trial [38, 40]	2	12
Targeted cohort e.g. low socioeconomic area [9, 41]	2	12

a Questions related only to primary qualitative papers *n* = 17.

### Data collection and sampling

Of the 16 studies conducted using interviews, the majority were semi-structured using interview guides (*n* = 13, 81%). Five of the studies (35%) used paired interviews to elicit different perspectives from the same patients at different time points in the surgical journey. Sample sizes varied between 5 and 39 participants. Recruitment was commonly through treating surgeons or clinicians (*n* = 7, 41%) or via purposive sampling of patients on a given waiting list (*n* = 6, 35%).

### Results of individual sources of evidence

Details of each included paper can be found in Table 2. A concise thematic summary of the results with exemplar quotes can be found in Table 3, with a more exhaustive list included as a Supplementary File.

**Table 2:** Details of the 21 included studies.

Surname year	Title	Country	Aim/objective of the study	Data collection methods	Study participants
Bur 2024 [21]	Patient experiences during the planned perioperative care pathway: an integrative review	Australia	This integrative review aimed to synthesize evidence about the patients’ experiences during the planned perioperative care pathway.	Integrative literature review	22 included studies, 6 orthopaedic, participants aged 13–85 years old
Longo 2023 [30]	Patients’ perceptions and experiences during the pre-admission phase for total hip replacement surgery: a qualitative phenomenological study	Italy	This study aims to explore patient experiences during preadmission for total hip replacement.	Semi-structured interviews	12 patients waiting total hip replacement, (7 female; 5 male), mean age 69 years
Gill 2019 [31]	Waiting in isolation: a qualitative patient researcher led study of the experience of patients waiting for elective surgery	Canada	This Patient and Community Engagement Research (PaCER) qualitative research study informs the larger project about the patient experience of waiting for elective surgery from time of being assessed as ready to treat or, as patients perceive it, in need of surgery for what has been diagnosed as a nonurgent medical condition until the surgery date.	Narrative interviews	18 participants 9 orthopaedics
Gustafsson 2010 [27]	In the hands of formal carers: older patients’ experiences of care across the perioperative period for joint replacement surgery	Sweden	To illuminate the meaning of care from all formal carers before, during, and after joint replacement surgery of the hip or knee.	Semi-structured interviews	12 completed paired interviews 6 men, 6 women aged 67–85 years 9 THR; 3 TKR
Webster 2013 [32]	Where is the patient in models of patient-centred care: a grounded theory study of total joint replacement patients	Canada	To explore the experiences of people scheduled for total hip or knee replacement, focusing on osteoarthritis as a chronic disease and how these experiences align (or not) with models of patient-centred care.	Semi-structured interviews	33 participants (17 female, 16 male) aged 38–79; 20 THR and 13 TKR
Urakcheeva 2025 [33]	Understanding patients’ emotional wellbeing, perceptions and concerns during an unprecedented deferment of arthroplasty: an Italian qualitative study	Italy	To explore the feelings, perceptions, concerns, and perceived quality of life of Italian orthopaedic patients whose elective arthroplasty was deferred due to COVID-19-related healthcare measures.	In-depth semi-structured interviews	26 orthopaedic patients (12 males, 14 females), aged 20–84 years, from North, Centre, and South of Italy
Tay Swee Cheng 2015 [34]	Factors relating to perioperative experience of older persons undergoing joint replacement surgery: an integrative literature review	Multiple countries	The primary aim of this review was to identify the factors relating to the perioperative experience of older persons undergoing primary total hip and knee replacement surgery resulting from osteoarthritis.	Integrative literature review	22 papers; 9 qualitative
Robling 2009 [35]	Time to talk? Patient experiences of waiting for clinical management of knee injuries	United Kingdom (Wales)	To explore patient experiences of waiting for specialist diagnosis and surgery for knee injuries at a UK centre.	Semi-structured, in-depth interviews	39 patients completed baseline interviews; 36 completed paired interviews. Adults aged 18–65 years; variation by gender and age.
Pillastrini 2025 [36]	Patients’ experience on waiting for spinal arthrodesis: a qualitative study	Italy	To explore the experiences, emotions, expectations, and health-related quality of life of patients while on the waiting list for lumbar spinal arthrodesis.	semi structured interviews	15 patients (10 female, 5 male), aged 23–80 years, awaiting lumbar spinal arthrodesis for the first time.
Parsons 2009 [28]	Living with severe osteoarthritis while awaiting hip and knee joint replacement surgery	United Kingdom	To explore the lived experiences of patients with severe osteoarthritis (OA) of the hip or knee joint while awaiting joint replacement surgery.	Unstructured, in-depth interviews	6 participants aged 60–76 years; 3 males; 3 females; 3 THR 3 TKR
Moffatt 2024 [40]	Exploring the experiences and perceptions of patients awaiting rotator cuff repair surgery: An integrated qualitative study within the POWER pilot and feasibility trial	United Kingdom	Explore experiences of adults awaiting rotator cuff repair surgery in the NHS.	semi-structured telephone interviews	20–12 males, 8 females, 49–81 years of age
Johnson 2014 [26]	Conceptualizing time before surgery: the experience of patients waiting for hip replacement	United Kingdom	To examine the experience of time within a longitudinal qualitative study of patients waiting for hip replacement.	Paired, in depth, semi-structured interviews	24: 13 men, 11 women aged 52–82 years
Jack 2022 [15]	Identifying and understanding the non-clinical impacts of delayed or cancelled surgery in order to inform prioritization processes: a scoping review	United Kingdom	To identify evidence related to the nature and prevalence of social and economic harm experienced by patients associated with delayed surgery.	Scoping literature review	21 articles: 4 reviews, 5 qual, 10 quant, 1 mixed, 1 commentary
Fowler Davis 2022 [41]	Understanding the needs and priorities of people living with persistent pain and long-term musculoskeletal conditions during the COVID-19 pandemic: a public involvement project	United Kingdom	To understand the experiences, needs and priorities of those from three underrepresented groups, whose musculoskeletal pain may have been exacerbated or had treatment delayed secondary to COVID-19.	Interviews, nominal group method	8 people with fibromyalgia 4 HCWs in musculoskeletal services 6 HCWs in aged care homes, no residents 7 elective orthopaedic surgery patients, 2 HCWs 12 people attended nominal group method
Engstrom 2017 [37]	Women’s experiences of undergoing total knee joint replacement surgery	Sweden	The aim of this study was to describe women’s experiences of undergoing total knee joint replacement surgery	Individual semi-structured interviews	5 women aged 62–84 years
Davis 2013 [38]	Exploring the care experience of patients undergoing spinal surgery: a qualitative study	United Kingdom	The aim of this study was to explore the healthcare experience of patients undergoing spinal surgery with a view to further understanding their needs and the issue that led to dissatisfaction.	2 focus groups	7 participants
Crick 2022 [39]	Waiting for infected hip revision surgery; exploring patient and families’ experience of waiting (PREWs)	United Kingdom	The aim of this study was to understand prosthetic joint infection (PJI) patients’ and their families’ experiences of waiting for revision surgery for hip infection and their perceptions of its impact.	Semi-structured interviews	5 participants: 4 male, 1 female Age 39–59+
Chan 2025 [9]	Patient experiences of waiting for orthopaedic care and priorities for ‘waiting well’: a qualitative study in a London NHS trust	United Kingdom	This study aimed to understand the experiences of patients waiting for orthopaedic treatment, and what would support people to live well while waiting, focusing on those in the most deprived areas.	Semi-structured interviews	7 patients interviewed from large NHS Trust 20–79 years old 2 female, 5 males 4 currently waiting, 3 already treated
Carr 2014 [25]	Time while waiting patients’ experiences of scheduled surgery	Canada	To explore patients’ experiences of time while waiting for three types of surgery with varied wait times: hip or knee replacement, shoulder surgery, and cardiac surgery.	Paired, semi-structured interviews	32 interviews (22 ortho), most 50–60 years. Aged 43–89 years
Carr 2009 [1]	Waiting for surgery from the patient perspective	Canada	The aim of this study was to perform a systematic review of the impact of waiting for elective surgery from the patient perspective, with a focus on maximum tolerance, quality of life, and the nature of the waiting experience.	Systematic literature review	27 original articles included
Carr 2017 [24]	Waiting for scheduled surgery: a complex patient experience	Canada	The aim of this study was to explore and understand experiences of wait time among patients awaiting scheduled orthopaedic or cardiac surgery.	Paired, semi-structured interviews	32, 16 men, 22 ortho, 16 cardiac, aged 43–89 years

**Table 3:** Resultant themes from the review with exemplar quotes.

Client: Waiting for surgery significantly impacts patients’ mental health and wellbeing	
Pain negatively impacts daily life activities	‘It changes your life, you don’t live anymore … making a move in the bed is a sacrifice, taking a shower is a sacrifice, putting on pants is a sacrifice …’ p.5 [30]
Waiting had negative effect on health	‘I think some days I get depressed. You’re feeling like, when is this going to get done?’ Table 4 [31]
Living a life on hold where the waiting list is like being in a ‘black hole’	‘The biggest problem for me is that I can’t do a lot of stuff. I go outside and walk for 5–10 minutes, and I have to sit down because of the pain. I can’t do anything. I can’t golf or do things with my kids. (because of hip pain.) It’s taken my life away’ Table 6 [31]
Life on a pause	‘I am going through these pains without having done anything yet, this time that is passing is time lost’ Table 2 [33]
Responses and coping processes	‘Well, there’s not a lot you can do sitting in a chair physically, I think, once you lose the physical side of your life. I think it drags you down mentally, and in turn you become caustic about things—you definitely become angry, you know?’ Box 4 [35]
Impact of persistent pain on physical and psychosocial functioning	‘I’m seriously discouraged, if I don’t get the surgery done I’ll end it all: I can’t live like this any longer’ p.86 [36]
Fear of the unknown and uncertainties	‘When I’ll wake up, will I be able to walk? I’m so scared.’ p.836 [36]
Impact of the unpredictability of the waiting time	‘You don’t know how long the wait will be. This weighs on you: you can’t schedule anything for sure.’ p.838 [36]
Experience of shoulder pain and pathway to treatment including sense of frustration and abandonment - long periods of waiting with no communication from the hospital	‘So I’ve heard nothing from the surgical team, I’ve heard no appointment, I’ve heard no … they just said I was on a waiting list, and that was it.’ Table 2, p.4 [40]
Unavoidable changes to use and passage of time in the lead up to surgery	‘Just stuck. I mean I haven’t been anywhere apart from the doctors. I managed to get to my daughter’s wedding in April and my friends funeral in May. But apart from that I just don’t go anywhere. I just can’t do it.’ p.129 [26]
Immediate impacts of waiting on patients’ wellbeing	‘I don’t like asking for help—this is a big thing for me. I don’t know, I feel a sense of embarrassment, shame, [wondering] if people are going to judge.’ p.7 [9]
Uncertainty	‘It gets me a little angry when you try [to] find out where you are on the list, and nobody can tell you’ p.294 [24]
Impact of living conditions	‘I consider myself a lucky guy, I had the choice in this as in other fields…Also lucky to have access to private insurance’ Table 2 [33]

**Intervention: Hospital communication and care while waiting.**	
Sharing of information is empowering;	‘A majority of studies within this review reported that perioperative information provided by health professionals was a significant component of the patient experience’ p.3891 [21]
Hospital systems and care coordination influence the patient experience.	‘Studies reported that patients experienced difficulty knowing the right person to contact who could answer questions preoperatively and would wait for return telephone calls’ p. 3893 [21]
Challenges with understanding the system and reasons for waiting	‘That what happens…if someone needs a hip, you get bumped. How do you know if I’m being bumped? That is a big issue. I don’t know what’s happening. I have no confidence in the (public health) system right now.’ Table 16 [31]
Living a life on hold where the waiting list is like being in a ‘black hole’	‘I’m getting no support while I wait. I want communication. They should communicate with me. I don’t know who will communicate with me’ Table 7 [31]
Who to turn to for guidance across the perioperative period	‘It was difficult to contact experts at the hospital for information and guidance while waiting for surgery.’ p.102 [27]
Patient perception of clinician and their roles	‘but I had another appointment [at hospital] and my husband turned around and said to them … “what do you intend to do in the mean time for her pain,” … “Oh,” he said, “that’s nothing to do with us,” he said, “that’s your own doctor [i.e., family doctor] and the painkillers” … So, basically their attitude was—"we can’t do anything yet.’ Box 5 [35]
The experience of waiting	‘The experience of waiting may depend on the quality and frequency of communication from patients’ clinical teams, and how people can use their time in the interim.’ p.5 [15]
Uncertainty	‘It gets me a little angry when you try [to] find out where you are on the list, and nobody can tell you’. p.294 [24]

System: Waiting is inevitable in public health.	
Impact of the unpredictability of the waiting time	‘You don’t know how long the wait will be. This weighs on you: you can’t schedule anything for sure.’ p.838 [36]
Acceptance of system	‘I don’t want to complain because I think you all work so hard and I’m only one of thousands of patients’. p.3 [39]
Time drags	‘As time has gone by the pain has increased, the hip has become more unstable, and, um, I have had to go into my doctor several different times to try different types of pain medication to try and alleviate the pain’. p.1676 [25]
Time drags	‘Once you are going to find out that you’re going to have surgery, then it gets [expletive] aggravating and long drawn out waiting for it. Not that I am scared of it. I could give a [expletive]. I would have it tomorrow, but it is just you can’t do nothing. You can’t work properly; you can’t plan nothing.’ p.1677 [25]
Time flies	‘[My condition] is teaching me patience, which I don’t have much of in some things. And if you can’t change it, then do your fitness the best you can. That is another thing you have to learn to put up with it until something gets done.’ p.1678 [25]
Time as usual	‘I think I get progressively more disabled with time. I think that happens to a certain extent. As time goes on, you get older.’ p.1679 [25]
Minimum acceptable waiting times according to patients	‘When MAWT was assessed in patients awaiting hip or knee replacement surgery, MAWT ratings were related to patient pain, loss of mobility, time needed to prepare, and severity at consultation.’ p.111 [1]
Opportunity	‘It [waiting] has made me really tune into the important things within [my] lifestyle … and what are [my] priorities.’ p.298 [24]
Challenges while waiting for surgery, personal responsibility, lack of support	‘The system is so overwhelmed that you have to advocate for yourself.’ Table 9 [31]
The impact of formal carers across the perioperative period, feeling worthy and respected by carers	‘Any criticism of the carers’ actions was deliberately avoided throughout the hospitalization period for surgery or rehabilitation. Bad experiences were accepted in silence so there was no risk of “biting the hand that feeds”.’ p.104 [27]
Resignation	‘Like I have no control. I’m not driving the train, you know. There’s a whole bunch of other things and other needs and other people and other commitments that are driving the train’ p.295 [24]
Patient perception of clinician and their roles	‘but I had another appointment [at hospital] and my husband turned around and said to them … ‘‘what do you intend to do in the mean time for her pain,’’ … ‘‘Oh,’’ he said, ‘‘that’s nothing to do with us,’’ he said, ‘‘that’s your own doctor [i.e., family doctor] and the painkillers’’ … So, basically their attitude was—‘‘we can’t do anything yet.’ Box 5 [35]

Outcomes: Patient needs while they wait for surgery.	
Challenges while waiting for surgery, personal responsibility, lack of support	‘But if he had a little group of people that you could maybe talk to that would be valuable…A little feedback as to advice …or suggestion (around what you might do while you’re waiting as your back is getting worse).’ Table 10 [31]
Living a life on hold where the waiting list is like being in a ‘black hole’	‘The biggest problem for me is that I can’t do a lot of stuff. I go outside and walk for 5–10 minutes, and I have to sit down because of the pain. I can’t do anything. I can’t golf or do things with my kids. (because of hip pain.) It’s taken my life away’ Table 6 [31]
Living a life on hold where the waiting list is like being in a ‘black hole’	‘I’m getting no support while I wait. I want communication. They should communicate with me. I don’t know who will communicate with me’ Table 7 [31]
Help, advice and support while awaiting surgery	‘… I feel very tired; I want to sleep a lot … I feel very depressed … you get frustrated because you can’t do things and actually I have got to say I just don’t want to do anything … I can’t be bothered and now that I know I am having the operation, I keep saying “I will leave that until I am better”. I don’t bother to do things any more …’ p.131 [28]
Impact on employment	‘Participants in four qualitative papers identified that time off work or adjustments to their work activity was necessary whilst waiting for surgery [17–20]. Furthermore, reports emerged of longer-term negative impacts on career pathway plans [18] and negatively altered relationships with work colleagues’ p.5 [15]
The need for better information and preparation	‘It was the length of time between each stage of the procedure… there was a length of time, so I forgot what I had been told really about the procedure.’ p.134 [38]
The need to be proactive	‘I am a great believer in proactivity, you know, find out as much as YOU can and do whatever YOU can to assist’ p.135 [38]
The need to be able to speak up and ask questions	‘ He was running about 3/4 of an hour late and he just wanted to get people in and out…I didn’t have a chance to ask any questions’ p.135 [38]
The need for human contact	‘I personally like the fact of actually saying to somebody, look this has happened, should I be worried or shouldn’t I?’ p.136 [38]
The need for continuity of care	‘I think probably what helped me with that is because of the fact that I had X (doctor) right from day one so we could build up a relationship between us which we did, I’ p.136 [38]
Regular contact with healthcare service	‘None of the questionnaire respondents made any recommendations about how the waiting period could be improved. However, regular contact for patients with the hospital was seen as important for “staying in touch” and perceived to help with the waiting experience, especially when things went wrong.’ p.5 [39]

### Synthesis of results

Themes generated from the qualitative research studies presented in this review were categorized and interpreted according to the QHOM [23, 29], see Fig. 2.

**Figure 2:**
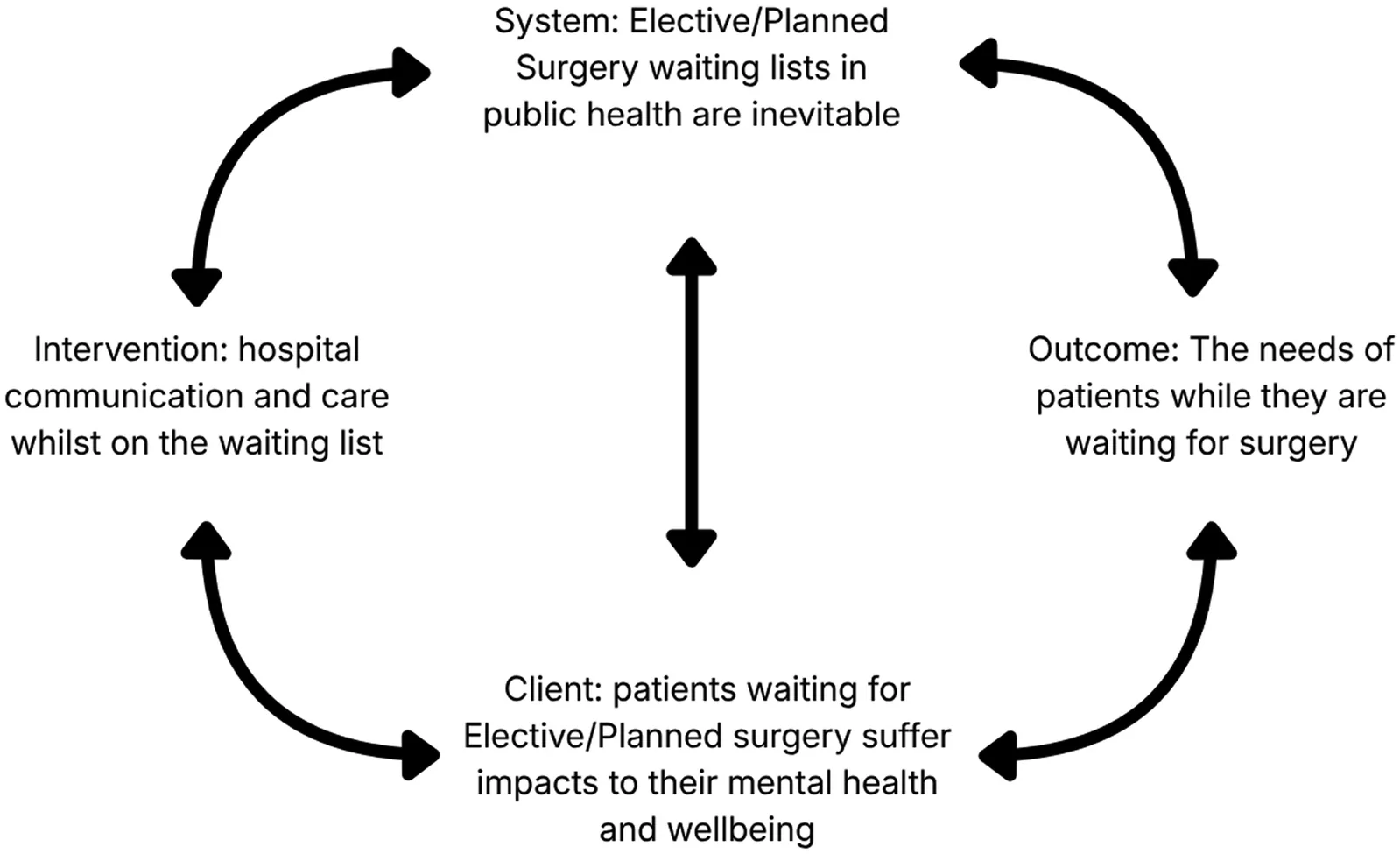
Results of scoping review explored within the QHOM which considers the client/patient and the system to have bidirectional influences upon the intervention and outcome, as well as upon each other.

#### Client: waiting for surgery significantly impacts patients’ mental health and wellbeing

The most pertinent theme described by participants in this review was the psychological impact imposed by the waiting period [1, 9, 24–26, 28, 30–38]. Many participants spoke of living a life on hold, *‘I go outside and walk for 5–10 minutes, and I have to sit down because of the pain. I can’t do anything. It’s taken my life away’* [31]. Worry, depression and fear about the uncertainty of the future and the potential for functional deterioration permeated many patient interviews [33, 34, 36, 38]. Others described a loss of control and autonomy in looking after themselves, and needing to depend on others [9, 24, 28, 34, 39]. Social isolation and immobility were identified as precursors to deterioration in mental health while waiting for surgery [15, 26, 37]: *‘I haven’t been anywhere apart from the doctors. I managed to get to my daughter’s wedding in April and my friends funeral in May. But apart from that I just don’t go anywhere’* [26]. This extended to the financial impost associated with not being able to work and being unable to make plans [15, 26, 28].

Pain and functional decline were the most frequent symptoms described by patients awaiting elective orthopaedic surgery, but it was the impact of pain on quality of life that caused the most distress [24, 25, 28, 30–32, 34–36, 40, 41], *‘It changes your life, you don’t live anymore … making a move in the bed is a sacrifice, taking a shower is a sacrifice, putting on pants is a sacrifice’* [30]. Once the decision for surgery was made, the waiting period also caused anger and frustration for patients [35]. The experience of delays and cancellations related to the COVID-19 pandemic pervaded studies published post 2020, where patients were both conciliatory about having to wait and concerned about getting sick while they waited [33].

#### Intervention: hospital communication and care while waiting

Patients’ experiences of communication from the healthcare services influenced their perception of care while they waited [1, 9, 15, 21, 27, 28, 34–36, 38–42]. Patients expressed a lack of contact with healthcare providers, *‘I’m getting no support while I wait. I want communication’* [31], and a lack of understanding of the surgical waiting list system [31]. There were questions about who to contact during the waiting period [21, 27], and confirmation that effective therapeutic relationships with healthcare workers are valued by patients during their uncertainty [15, 21, 41]. Concerns about care on the waiting list also stemmed from worry about physical deterioration and a lack of guidance on management of pain in the interim [31, 35, 41]. Patients felt that their support needs increased as a consequence of waiting, including medical management, practical help and information to allay anxiety [9, 38]. Several studies highlighted how patients felt lost in the system, invisible, like they’d fallen through the gap [31, 35, 40]. Furthermore, some patients just wanted honest information from healthcare staff: *‘I kept waiting for four years, being led like a goose chase. Just be honest with the timing, just tell us’* [9]. Patients also appreciated healthcare workers, *‘.you all work so hard and I’m only one of thousands of patients’* [39], although one study which also included patient experience of hospitalization for their surgery did highlight that patients were not necessarily forthcoming with feedback, *‘so there was no risk of “biting the hand that feeds’’’* [27].

#### System: waiting is inevitable in public health

Ideally, the solution to lengthy waiting times is treating more patients to shorten the list; however, patients in most studies accepted that waiting lists were inevitable [1, 24, 35, 39]. Patients considered the dynamic nature of healthcare and its limitations and acknowledged that other patients could be in a worse state of health than themselves [24, 31, 33, 35, 39, 41]; *‘You sit there and you think and think how lucky you are and how unlucky they are*’ [24]. Additionally, the concept of time was discussed both in the context of existing in a state of worsening health [25, 26] and recognizing the opportunity to prepare for the upcoming surgery [15, 24]. The ability of patients to cope while waiting was variable [9, 24, 28, 33–35, 40, 41] and influenced by a number of factors, especially socioeconomic opportunity, where two studies suggested that vulnerable cohorts within the population were inequitably impacted by the length of time on the list [9, 34].

#### Outcomes: patient needs while they wait for surgery

Patients clearly articulated gaps in healthcare provision while they waited for surgery [9, 15, 27, 28, 31, 32, 34–36, 38–41]. The resounding need was for improved communication between the hospital and patient. A review by Jack *et al.* found that *‘the experience of waiting may depend on the quality and frequency of communication from patients’ clinical teams, and how people can use their time in the interim’* [15]. Examples of how the communication could occur varied from regular phone contact from clinicians [39] to opportunities for peer group support from other patients [36], and care continuity throughout the waiting period [35, 38]. Several studies described the need for healthcare workers to understand patients’ personal circumstances in the context of waiting, including the impact on work and finances [15], and to be heard and respected [27, 38]. Several patients saw the waiting period as a time for preparation and considered their own obligations in this process [24, 25, 38, 41].

## Discussion

### Statement of principal findings

This qualitative scoping review sought to illuminate the patient perspective of waiting for elective orthopaedic surgery. The findings highlight that waiting presents a range of challenges, concerns and impacts upon patient health and wellbeing, extending beyond the initial indications for surgery [21]. While waiting times vary nationally and internationally, ongoing healthcare support is essential to patients during this period.

The QHOM provides a useful lens to interpret these review findings by making visible the dynamic interactions between system factors, patient experiences and care processes during the waiting period. Rather than functioning in a linear pathway, the waiting list can be understood as a series of feedback loops in which system-level conditions, particularly gaps in communication, unclear care ownership, and limited access to support, shape patient experience (client), which in turn influences health outcomes and readiness for surgery. For example, fragmented communication contributes to uncertainty and psychological distress, which may exacerbate physical deterioration and reduce patients’ capacity to engage in preoperative optimization. Conversely, interventions that prioritize continuity, timely information, and psychosocial support have the potential to interrupt these negative cycles by improving patient experience and stabilizing health status while waiting [9]. Viewed through this model, the waiting period emerges as a modifiable phase of care where targeted, patient-centred interventions could influence both immediate wellbeing and downstream surgical outcomes. This reinforces the need to move beyond a purely capacity-driven view of waiting lists towards a more integrated model of care that actively supports patients throughout the preoperative journey [43].

### Strengths and limitations

Although patient experiences have been explored using Patient Reported Outcome and Experience Measures, the quantitative nature of this data did not lend itself to the analysis undertaken in this review and were therefore excluded. In the almost two decades since the review by Carr *et al.* was published, limited research has examined patient experience of waiting for elective surgery, despite continued growth in waiting lists [1]. The absence of literature from low- and middle-income countries (LMICs) in this review may reflect the fact that less than 5% of surgical procedures worldwide occur in these regions [44]. Moreover, resource constraints in LMIC health systems face challenges in funding for surgical training, surgical safety and research, which may limit their academic contributions [45]. Additional methodological constraints specific to scoping reviews include a lack of quality appraisal, contextual limitations and interpretive bias in the synthesis of results.

### Interpretation within the context of the wider literature

Communication needs during the perioperative period have not changed in previous decades, with research demonstrating the benefit of nurse-led communication to support patients waiting for elective surgery [46]. The COVID-19 pandemic further highlighted the mental health impacts associated with delayed surgery, isolation and uncertainty [33]. In a review examining the psychological burden of waiting for transplant, cancer surgery and outpatient care during COVID-19, patients identified several areas for improvement, even where waiting times could not be reduced [47]. These included the need for acknowledgement and empathy from health professionals regarding the mental health impacts of waiting; regular updates about delays, waiting list position, prioritization criteria and an anticipated timeline; and access to supportive interventions such as peer support and mental health services [47]. Similarly, a recent study with of patients awaiting cardiac surgery identified communication and regular updates from the hospital as key as areas for improvement [48].

The surgical liaison nurse role in public health was created specifically to address many of these challenges by coordinating perioperative care and providing education and reassurance to patients and families [49]. Despite longstanding recognition of these needs, consistent implementation remains challenging. Factors such as workforce constraints [50] limit the capacity for proactive communication, while the increasing digitization of healthcare presents both opportunities and challenges for maintaining effective patient–provider communication [51]. Nevertheless, the surgical liaison nurse has been shown to play an important role in bridging the divide between primary and specialist care for patients awaiting planned surgery and may present an underutilized resource in this context [52].

### Implications for policy, practice, and research

This synthesis identified two interrelated system-level issues that have direct implications for the design of elective surgery pathways. First, the waiting period poses a substantial but largely unaddressed psychosocial burden, where patients experience escalating pain, loss of function, and emotional distress with limited structured support. Second, patients consistently describe fragmented and opaque communication across the care continuum, contributing to a sense of uncertainty, loss of control, and feeling ‘lost’ between primary and specialist care. Importantly, these issues do not operate in isolation: inadequate communication exacerbates psychosocial distress, while unmet support needs amplify patients’ perceptions of system neglect. Together, these findings suggest that waiting lists are not simply logistical challenges of capacity, but reflect a lack of coordinated, patient-centred care and communication during the preoperative period. Addressing these gaps requires a shift from viewing waiting as administrative delay to recognizing it as an active phase of care that warrants structured communication, continuity, and support. Several papers offer suggestions to improve communication, such as quarterly automated SMS check-ins with patients on the waiting list [9], and identify that reform to planned surgery needs to include stronger links between primary and tertiary care in the perioperative period [53]. Future research could focus on implementation of practical, scalable solutions.

## Conclusion

Elective surgery waiting lists are likely to be a topic for further public scrutiny in the years ahead. Remedial work is underway to reduce the length of waiting and enhance preparation of patients for their surgery including prehabilitation. Reframing the waiting list time period as an active phase of care, rather than a passive delay provides scope to address key concerns outlined through patient experience and described in this review. Actionable system-level targets include improving communication between patients and their healthcare providers, fostering continuity of care and addressing psychosocial needs. Understanding what matters most to patients waiting for surgery is essential to deliver effective patient-centred care in public health.

## Supplementary Material

mzag164_Supplementary_Data

## Data Availability

The search strategy employed for this review is available for download in Supplementary Materials, as is the data extraction template and the PRISMA-ScR Checklist.
