# Supplementary material for: Waiting for elective orthopaedic surgery: a qualitative scoping review of the patient experience
Source: Int J Qual Health Care. 2026 Oct 10;38(4):mzag164. doi: 10.1093/intqhc/mzag164 (PMC13690467; doi:10.1093/intqhc/mzag164)
Supplement: Supplementary file 1 [file mzag164_supplementary_data.zip › Data Extraction Template.docx]

**General information**

- Title
- Lead author surname and year
- Primary author contact details
- Country in which the study was conducted
- Was there documentation of ethics approval in study? Participant consent etc.

1. Yes
2. Other

**Introduction**

- Gap / Problem statement (description/significance of problem studied)
- Aim / Objective of the study

**Methods**

- Qualitative approach, (eg. grounded theory, phenomenology, case study); and research paradigm (eg. positivist, interpretivist)

**Data Collection**

- Theoretical models / underpinnings (eg. Quality Health Assessment, Theory of change)
- How was the data collected? (eg. F2F interviews / focus groups)
- Instruments/technologies used- (eg. interview guides, questionnaires, audio recording,)

**Participant (Population)**

- Number, characteristics eg. gender, age
- Cohort:

1. Ortho
2. Ortho + Other speciality

- Sampling / Recruitment Strategy (eg. how (phone/flyer) and why (inclusion criteria) research participants were selected, criteria for deciding when no further sampling is required

**Data Analysis**

- How was the data processed? (eg. recordings transcribed, who entered data, how was it verified, who did data coding and how? What was done to preserve anonymity?)
- How was the data analysed? (eg. using a specific paradigm? What was the process for identifying/developing themes/inferences? Who was involved in the data analysis?)
- How did the paper discuss researcher characteristics and reflexivity - things that may influence the research eg. personal attributes, qualifications, relationship to participants, assumptions?
- Strategies for Ensuring Trustworthiness in Qualitative Research:

1. Prolonged Engagement: Spend adequate time in the field, building rapport with participants to understand their perspectives deeply.
2. Reflexivity: Acknowledge personal biases and preconceptions throughout the research process.
3. Triangulation: Employ multiple data sources or methods (e.g., interviews, observations, documents) to cross-verify findings.
4. Thick Descriptions: Provide detailed contextual information to enable readers to assess the transferability of findings.
5. Sampling Strategies: Clearly articulate the sampling process and criteria to justify the potential transferability of the findings.
6. Methodological Documentation: Detail the research procedures and decisions made during the study.
7. Audit Trails: Keep an audit trail of research decisions, changes, and data analysis processes to ensure traceability.
8. Peer Debriefing: Engage with colleagues or experts to review interpretations and findings, minimizing researcher bias.
9. Member Checking: Allow participants to review and confirm the accuracy of the findings to enhance confirmability.
10. Reflexive Journaling: Maintain a reflective journal documenting personal thoughts, biases, and reflections throughout the study.
11. Other

**Results & Discussion**

**Context**

- How does this study fit into the context of the review (eg. perioperative period, public health, orthopaedic surgery)

**Concept**

- How does this study relate back to the concept of patient experience of waiting for elective orthopaedic surgery? (eg. interviews were used to extract patient experience, outcomes included distress with waiting times etc.)
- What are the main findings, themes?
- Is there evidence within the paper of links to empirical data? Eg. quotes from participants, field notes)
- Did the study acknowledge any limitations? if so, what were they?
- Were there any conflicts of interest identified? If so, what ?
- What was the source of funding for this study, and what was the role of the funders in data collection, interpretation and reporting?
