# Supplementary material for: Waiting for elective orthopaedic surgery: a qualitative scoping review of the patient experience
Source: Int J Qual Health Care. 2026 Oct 10;38(4):mzag164. doi: 10.1093/intqhc/mzag164 (PMC13690467; doi:10.1093/intqhc/mzag164)
Supplement: Supplementary file 1 [file mzag164_supplementary_data.zip › ScR Manuscript Full Exemplar Quotes Table for Supp.docx]

**Waiting for elective orthopaedic surgery: A qualitative scoping review of the patient experience.**

Supplementary Table: Resultant themes of each included paper with exemplar quotes

| **AUTHOR, YEAR** | **title** | **theme** | **EXEMPLAR QUOTE** |
| --- | --- | --- | --- |
| Bur   2024^21^ | Patient experiences during the planned perioperative care pathway: An integrative review | Sharing of information is empowering; | "A majority of studies within this review reported that perioperative information provided by health professionals was a significant component of the patient experience" p.3891   "Studies reported that patient's satisfaction with the perioperative care pathway was evaluated through a lens of anxiety and fear." p.3892 |
|  |  | Interpersonal relationships are valued by patients; | "Studies identified that when patients were asked by health professionals to repeat essential information about medical history and allergies, it reduced their opportunity to talk about their emotional wellbeing, which resulted in a feeling that health professionals were not listening to them" p. 3892 |
|  |  | Hospital systems and care coordination influence the patient experience. | "Studies reported that patients experienced difficulty knowing the right person to contact who could answer questions preoperatively and would wait for return telephone calls" p. 3893 |
| Longo  2023^30^ | Patients' Perceptions and Experiences during the Pre-Admission Phase for Total Hip Replacement Surgery: A Qualitative Phenomenological Study | Surgery generates mixed feelings | “I hope to improve. I’m optimistic; I’ve forgotten how to walk without feeling  pain, without limping, it’s something I see far. I want to be what I was . . . ” p.4  "I’ve been fighting with this hip for years, now I’ve really reached the limit . . . ” p.5 |
|  |  | Pain negatively impacts daily life activities | "It changes your life, you don’t live anymore . . . making a move in the bed is a sacrifice, taking a shower is a sacrifice, putting on pants is a sacrifice . . . ” p.5 |
|  |  | Pain requires personal strategies to be alleviated | "“I sleep badly, with a pillow between my legs and always in the same position, I cannot turn around because I have continuous pain even at night . . . " p.5 |
| Gill   2019^31^ | Waiting in isolation: A qualitative patient researcher led study of the experience of patients waiting for elective surgery | Waiting had negative effect on health | "SN270 The worst was the progressive pain and dealing with that on a daily basis. It kept getting worse" Table 2   "The symptoms are getting worse, they are not getting better, and they are not staying the same. While I’m waiting for surgery, I’m getting worse." Table 3   "I think some days I get depressed. You’re feeling like, when is this going to get done?" Table 4 |
|  |  | Living a life on hold where the waiting list is like being in a 'black hole' | "Knowing that I will have surgery sometime this year, I want to reserve my sick days for when I have the surgery. I get probably about a week sick time." Table 5   "The biggest problem for me is that I can’t do a lot of stuff. I go outside and walk for 5-10 minutes, and I have to sit down because of the pain. I can’t do anything. I can’t golf or do things with my kids. (because of hip pain.) It’s taken my life away" Table 6   "I’m getting no support while I wait. I want communication. They should communicate with me. I don’t know who will communicate with me" Table 7 |
|  |  | Challenges while waiting for surgery, personal responsibility, lack of support | "The system is so overwhelmed that you have to advocate for yourself." Table 9  "But if he had a little group of people that you could maybe talk to that would be valuable…A little feedback as to advice …or suggestion (around what you might do while you’re waiting as your back is getting worse)." Table 10 |
|  |  | Challenges with understanding the system and reasons for waiting | "I didn’t talk to him, but I talked to his staff, and they said that it is because of the line-up of people ahead of you. They go in sequence. There are too many clients" Table 13   "That what happens…if someone needs a hip, you get bumped. How do you know if I’m being bumped? That is a big issue. I don’t know what’s happening. I have no confidence in the (public health) system right now." Table 16 |
| Gustafsson   2010^27^ | In the hands of formal carers: Older patients' experiences of care across the perioperative period for joint replacement surgery | Who to turn to for guidance across the perioperative period | "It was difficult to contact experts at the hospital for information and guidance while  waiting for surgery. ". P.102 |
|  |  | Formal carers control the process of care | "The doctor at primary healthcare said, ‘there is no need for a letter of referral for surgery because your knee is just fat and swollen’. But I got an X-ray anyway.... then I was told that both knee and hip were worn out, but nothing could be done about it" p.103 |
|  |  | The impact of formal carers across the perioperative period, feeling worthy and respected by carers | "Any criticism of the carers’ actions was deliberately avoided throughout the hospitalization period for surgery or rehabilitation. Bad experiences were accepted in silence so there was no risk of ‘‘biting the hand that feeds’’." p.104 |
| Webster   2013**^32^** | Where is the patient in models of patient-centred care: a grounded theory study of total joint replacement patients | Patient experience of multiple joint symptoms and multiple chronic conditions | "… the frustrating thing is one doctor is telling you, you have to lose weight and the next [specialist is] saying don’t move, you have to sit with your leg up. Well, who do you listen to? It’s frustrating" p.4 |
|  |  | Experience of osteoarthritis as a chronic disease | "It just was very difficult to get through the workday. I mean, I was put on modified duties for such a long period of time because, well, my surgeon at the time… didn’t feel that I could ever return to regular duty … because of my knees being so bad…." p.4 |
|  |  | Concerns related to prescription pain management | "I don’t like medication, so I try to stay away from it. I try to manage [my OA pain] with as few pain killers as possible.” p.5 |
|  |  | Misalignment between patient and clinician understanding of readiness for surgery | "I was already taking prescribed anti-inflammatories, and I had a prescription for Tylenol 3 to take PRN so yeah, I was disappointed that they weren’t going to be doing anything for quite a while. And I thought well maybe I’m just not experiencing enough discomfort, and I had a fair way to go" p.5 |
| Urakcheeva   2025**^33^** | Understanding patients' emotional well-being, perceptions and concerns during an unprecedented deferment of arthroplasty: an Italian qualitative study | Negative emotional state due to deferment and lockdown | "‘I prayed that nothing acute would occur to me (during the waiting period)’ " Table 2 |
|  |  | Life on a pause | "‘I am going through these pains without having done anything yet, this time that is passing is time lost’" Table 2 |
|  |  | Deterioration in quality of life | "We have all experienced a deterioration in the quality of life’ " table 2 |
|  |  | Inner resources and attitudes for coping | "‘The only thing that sustained me was hope that something would improve and that we would go to surgery." Table 2   "I am a fairly positive person. That helped me. I have always thought things begin and then end too, like the cycle of life’ " Table 2 |
|  |  | Impact of living conditions | "I consider myself a lucky guy, I had the choice in this as in other fields…Also lucky to have access to private insurance’ " Table 2 |
|  |  | The lockdown paradox | "The fact of being sick and not being able to walk and having to stay at home anyway because of the lockdown—it honestly weighed less on me’ " table 2 |
| Tay Swee Cheng   2015^34^ | Factors relating to perioperative experience of older persons undergoing joint replacement surgery: an integrative literature review | Waiting time; Pain and disability; Mental health; race/ethnicity/age/gender; Body image; Coping and social support; Patient education; Care continuity | no quotes in paper |
| Robling   2009^35^ | Time to talk? Patient experiences of waiting for clinical management of knee injuries | Patient responses to system processes, | " It’s been cancelled. This is the third appointment … he sort of got, came home from the little job that he were doing, and they cancelled it about an hour before he was due to go up there." Box 1 |
|  |  | Perceived impact of waiting | "It’s the same every day, and it’s sort of curtailed everything, and to wait 3 years—well it’s been horrendous to be honest." Box 3 |
|  |  | Responses and coping processes | "Well, there’s not a lot you can do sitting in a chair physically, I think, once you lose the physical side of your life. I think it drags you down mentally, and in turn you become caustic about things—you definitely become angry, you know? ‘" Box 4   " I just feel so sorry for some of the older people, because they’re treated horrendous I think. Not too bad for me, I’m still young, and I can get around, but some of them have been waiting so long. It’s awful—the waiting is the worst thing." Box 4 |
|  |  | Patient perception of clinician and their roles | "but I had another appointment [at hospital] and my husband turned around and said to them … ‘‘what do you intend to do in the mean time for her pain,’’ … ‘‘Oh,’’ he said, ‘‘that’s nothing to do with us,’’ he said, ‘‘that’s your own doctor [i.e., family doctor] and the painkillers’’ … So, basically their attitude was—‘‘we can’t do anything yet." Box 5 |
| Pillastrini   2025^36^ | Patients' experience on waiting for spinal arthrodesis: a qualitative study | Impact of persistent pain on physical and psychosocial functioning | "“I’m seriously discouraged, if I don’t get the surgery done I’ll end it all: I can’t live like this any longer.” p.86 |
|  |  | Fear of the unknown and uncertainties | “When I’ll wake up, will I be able to walk? I’m so scared.” p.836 |
|  |  | Hope and regaining normality | “I hope everything is going to be alright and I want to believe it will. Because I want to go back to live as I did before.” p.838 |
|  |  | Impact of the unpredictability of the waiting time | "You don’t know how long the wait will be. This weighs on you: you can’t schedule anything for sure.” p.838 |
|  |  | Need for better communication and information on the care pathway | "I look at my friends, and I have this problem and I am different from them. Sometimes I wish I could talk to people who have already experienced this.”" p. 839 |
| Parsons   2009^28^ | Living with severe osteoarthritis while awaiting hip and knee joint replacement surgery | Living and coping with pain | ". . . So, the pain . . . honestly it is so intense . . . it is like someone is stabbing you! It really is excruciating sometimes . . . I mean, I cannot describe it any other way . . .’ p.128 |
|  |  | Loss of independence | "‘. . . When bending, it does hurt and I become desperate for some help . . . I never leave the house on my own – my husband is always with me . . .’" p.129 |
|  |  | Coping with everyday activities | "When I get down low to switch on the video I have to roll over on the floor to get back   up and hold onto the furniture to pull up" p.129 |
|  |  | How others see me | ". . . There’s something about having a stick, people judge you, you see. I think it makes you look older (having a stick) . . . I wouldn’t like people to think that I didn’t need one . . .’" p.130 |
|  |  | Help, advice and support while awaiting surgery | "‘. . . I feel very tired; I want to sleep a lot . . . I feel very depressed . . . you get frustrated because you can’t do things and actually I have got to say I just don’t want to do anything . . . I can’t be bothered and now that I know I am having the operation, I keep saying “I will leave that until I am better”. I don’t bother to do things any more . . .’" p.131 |
|  |  | Effect upon family, friends and helpers | ". . . I mean your family help if they can, but they have got their own lives to lead . . . but I try not to bother them for anything unless it’s urgent . . ." p.131 |
| Moffatt   2024^40^ | Exploring the experiences and perceptions of patients awaiting rotator cuff repair surgery: An integrated qualitative study within the POWER pilot and feasibility trial | Experience of shoulder pain and pathway to treatment including sense of frustration and abandonment - long periods of waiting with no communication from the hospital | "I paid private, just basically to find out if there was anything, ’cause obviously from January to September, I’ve been in like total agony… But, I’ve just been waiting, thinking that I’m going to get the operation date and obviously it never arrived” p.4  "So I’ve heard nothing from the surgical team, I've heard no appointment, I’ve heard   no … they just said I was on a waiting list, and that was it.” table 2 p.4  "You’re just left completely waiting and not knowing." table 2 p.4 |
| Johnson   2014^26^ | Conceptualising time before surgery: the experience of patients waiting for hip replacement | Unavoidable changes to use and passage of time in the lead up to surgery | "Just stuck. I mean I haven't been anywhere apart from the doctors. I managed to get to my daughter's wedding in April and my friends funeral in May. But apart from that I just don't go anywhere. I just can't do it." p.129  "I was in the village choir. Which I gave up because I couldn't sit still for very long … so that's some of our social life wiped out". p.129 |
|  |  | Time in the context of healthcare, fluid, dynamic, complex | "Yeah you can never make any plans, and you're just in limbo really e what if?" p.131 |
| Jack   2022^15^ | Identifying and Understanding the Non-Clinical Impacts of Delayed or Cancelled Surgery in Order to Inform Prioritisation Processes: A Scoping Review | Impact on employment | " Participants in four qualitative papers identified that time off work or adjustments to their work activity was necessary whilst waiting for surgery [17–20]. Furthermore, reports emerged of longer-term negative impacts on career pathway plans [18] and negatively altered relationships with work colleagues" pg.5 |
|  |  | Impact on social function and leisure activities |  |
|  |  | Impact on patients' finances |  |
|  |  | The experience of waiting | ". The experience of waiting may depend on the quality and frequency of communication from patients’ clinical teams, and how people can use their time in the interim." pg.5 |
|  |  | Potential patient assessment tools for future utilisation (last theme is deductive and agreed upon by all authors) |  |
| Fowler Davis   2022^41^ | Understanding the Needs and Priorities  of People Living with Persistent Pain and Long-Term Musculoskeletal Conditions during the COVID-19 Pandemic-  A Public Involvement Project | Understanding pain and beliefs about elective surgery and pain | "Have been associated with worse health perceptions, deterioration in quality of life and raised anxiety, as well prolonging sick leave and loss of income." p.7    "Most people waiting for elective surgery also feel resigned to having pain. This is because pain is often normalised as an inevitable aspect of waiting for surgery and they therefore regard it as something to be tolerated." p.7 |
|  |  | Impact of pain | "Despite stoicism and pain tolerance, pain can have a major impact on peoples’ daily  lives." p.7 |
|  |  | Communication with healthcare professionals | "Seeing professionals on a one-to-one basis is reassuring, and generic mailings  or information are no substitute for this." p.8 |
|  |  | Self-managing pain while waiting | "Information is power—letting people know early in their wait what they can do to manage their pain would be best." p.8 |
|  |  | Differences in experiences for different patients | "Individuals’ experiences become worse over time, with pain affecting sleep or activities of daily living, particularly with older patients." p.8 |
| Engstrom   2017^37^ | Women's Experiences of Undergoing Total Knee Joint Replacement Surgery | Time before surgery | "So, the wait is long, as you often have to be on sick leave and go back and forth to doctor’s appointments." p.89    "I did not dare to go out. I mean, you become afraid of slipping on the snow as you feel unstable in the knee joint. " p.89    "There were several people there for surgical assessment and many were, of course,  much worse than I am". p.90 |
| Davis   2013^38^ | Exploring the care experience of patients undergoing spinal surgery: a qualitative study | The need for reduced waiting times | "I personally found the waiting time quite stressful . . . but I  was in great pain" p.133 |
|  |  | The need for better information and preparation | "It was the length of time between each stage of the procedure. . . there was a length of time, so I forgot what I had been told really about the procedure." p.134 |
|  |  | The need to be proactive | "I am a great believer in proactivity, you know, find out as much as YOU can and do whatever YOU can to assist" p.135 |
|  |  | The need to be able to speak up and ask questions | " He was running about 3/4 of an hour late and he just wanted to get people in and out...I didn’t have a chance to ask any questions" p.135 |
|  |  | The need to feel safe | " I think for all of us, surgery is a major thing and it  is the vulnerability of us after the surgery, it is so significant  that one feels that one is safe" p.135 |
|  |  | The need to be treated with dignity and respect |  |
|  |  | The need for ongoing care and support | "I brought somebody with me (to my consultation), and I had them taking notes which helped" p.135 |
|  |  | The need for human contact | "I personally like the fact of actually saying to somebody, look this has happened, should I be worried or shouldn’t I?" p.136 |
|  |  | The need for continuity of care | "I think probably what helped me with that is because of the fact that I had X (doctor) right from day one so we could build up a relationship between us which we did, I" p.136 |
| Crick   2022^39^ | Waiting for infected hip revision surgery; exploring patient and families' experience of waiting (PREWs) | Acceptance of system | " “I don’t want to complain because I think you all work so hard and I’m only one of thousands of patients”. " p.3 |
|  |  | Emotional/social/physical impact | "The experience of waiting and the time from referral to surgery was also affected by pain. Most respondents reported that their pain had increased from moderate to severe while waiting" p.4 |
|  |  | Impact on family | "There was a bit of a long wait, and my family were getting very fretful. My son and daughter have both been very good and supporting and chasing up.” p.4 |
|  |  | Regular contact with healthcare service | "None of the questionnaire respondents made any recommendations about how the waiting period could be improved. However, regular contact for patients with the hospital was seen as important for “staying in touch” and perceived to help with the waiting experience, especially when things went wrong." p.5 |
| Chan  2025^9^ | Patient experiences of waiting for orthopaedic care and priorities for 'waiting well': a qualitative study in a London NHS trust | Immediate impacts of waiting on patients’ wellbeing | "“I don’t like asking for help – this is a big thing for me. I don’t know, I feel a sense of embarrassment, shame, [wondering] if people are going to judge. " p.7 |
|  |  | Long term consequences of extended wait time | "I don’t know if I might have been doing other things. You can only mourn what you know you have lost. That is the bigger loss. I am running out of years. P.8   "All the years, the other knee was compensating for it. I don’t want to get to the stage where I need the left knee done.” p.8   "I feel bloody angry that I have waited this long. My hope in thinking that I am going to be okay has gone down. " p.9 |
|  |  | Increased support needs as a consequence of waiting | "The painkillers are strong. I am like a zombie if I take them in the day. I can’t manage unless I am at home and have someone to take care of me. Literally, I can’t move around. " p.9   "I kept waiting for four years, being led like a goose chase. Just be honest with the timing, just tell us." p.10 |
|  |  | Co-design workshop generated 4 ideas: 1. check=in text, letter or call 2. physiotherapy invite letter 3. Coping toolkit. 4. Patient-centred staff training | "The first idea was to send a text message or letter to patients on the waiting list every three months which acknowledges how long the patient has been waiting and signposts to relevant support " p.11 |
| Carr   2014^25^ | Time while waiting patients' experiences of scheduled surgery | Time drags | "As time has gone by the pain has increased, the hip has become more unstable, and, um, I have had to go into my doctor several different times to try different types of pain medication to try and alleviate the pain" p.1676   "Once you are going to find out that you’re going to have surgery, then it gets [expletive] aggravating and long drawn out waiting for it. Not that I am scared of it. I could give a   [expletive]. I would have it tomorrow, but it is just you can’t do nothing. You can’t work properly; you can’t plan nothing." p.1677 |
|  |  | Time flies | "[My condition] is teaching me patience, which I don’t have much of in some things. And if you can’t change it, then do your fitness the best you can. That is another thing you have to learn to put up with it until something gets done." p.1678 |
|  |  | Time as usual | "I think I get progressively more disabled with time. I think that happens to a certain extent. As time goes on, you get older." p.1679 |
| Carr   2009^1^ | Waiting for surgery from the patient perspective | Minimum acceptable waiting times according to patients | "When MAWT was assessed in patients awaiting hip or knee replacement surgery, MAWT ratings were related to patient pain, loss of mobility, time needed to prepare, and severity at consultation." p.111 |
|  |  | The relationship between length of wait and HRQOL | "Patients placed on wait lists for hip or knee replacement surgery reported high psychological distress and poorer HRQOL than the population norm. This was especially the case for female patients and patients from lower socioeconomic groups." p.112 |
|  |  | Understanding the nature of the waiting experience from the patient standpoint | "Another study of 18 patients and their lived experience while waiting for hip or knee replacement surgery revealed one paradigm case and six themes. The paradigm case embodied the patient reality of a life in “no man’s land”, where uncertainty and loss of dignity prevailed. The commonality in all cases was waiting to return to a more normal life" p.115  "There was a lack of understanding of the waiting list process, and a further lack of communication from the hospital regarding patient position on the list and possible length of wait time." p.115 |
| Carr   2017^24^ | Waiting for scheduled surgery: A complex patient experience | Restriction | "Well, you’re waiting so far already, and it is frustrating … um, it also diminishes you … because you can’t do things that you have done all your life" p.294 |
|  |  | Uncertainty | "It gets me a little angry when you try [to] find out where you are on the list, and nobody can tell you" p.294 |
|  |  | Resignation | "Like I have no control. I’m not driving the train, you know. There’s a whole bunch of other things and other needs and other people and other commitments that are driving the train" p.295 |
|  |  | Coping with waiting | "You sit there and you think and think how lucky you are and how unlucky they are. Naturally your heart goes out [to] somebody worse than you are. " p.297 |
|  |  | Opportunity | "It [waiting] has made me really tune into the important things within [my] lifestyle … and what are [my] priorities. " p.298 |
