# Supplementary material for: Waiting for elective orthopaedic surgery: a qualitative scoping review of the patient experience
Source: Int J Qual Health Care. 2026 Oct 10;38(4):mzag164. doi: 10.1093/intqhc/mzag164 (PMC13690467; doi:10.1093/intqhc/mzag164)
Supplement: Supplementary file 1 [file mzag164_supplementary_data.zip › Search strategies.docx]

The search was last run 10^th^ June 2025 across the following databases:

- Ovid MEDLINE® ALL <1946 to June 06, 2025>
- Embase (Ovid) <1974 to 2025 June 06>
- Ovid Emcare <1995 to 2025 Week 22>
- CINAHL Complete (Ebsco)

Qualitative search filters from Canada's Drug Agency Search Filters Database was used for MEDLINE, Emcare and CINAHL:<https://searchfilters.cda-amc.ca/list?q=&p=1&ps=20&topic_facet=qualitative%20studies%20000000%7CQualitative%20studies>

The qualitative search filter for MEDLINE was adapted for Embase (Ovid).

Search conducted by: Mina Nichols-Boyd, Clinical Librarian, Austin Health Sciences Library.

Search peer reviewed by: Paula Todd, Academic Services Lead, Monash University Library.

**Database:**
Ovid MEDLINE(R) ALL <1946 to June 06, 2025>

| **#** | **Query** | **Results from 9 Jun 2025** |
| --- | --- | --- |
| 1 | Waiting Lists/ | 14,742 |
| 2 | Time-to-Treatment/ | 11,380 |
| 3 | Time Factors/ | 1,252,671 |
| 4 | (wait* adj3 (list* or time*)).ti,ab,kf. | 37,833 |
| 5 | ((delay* or time* or wait*) adj3 (surg* or operati* or procedure* or factor* or treatment*)).ti,ab,kf. | 343,631 |
| 6 | preparation list*.ti,ab,kf. | 9 |
| 7 | or/1-6 | 1,598,148 |
| 8 | elective surgical procedures/ or orthopedic procedures/ or arthroplasty, replacement, hip/ or arthroplasty, replacement, knee/ | 112,827 |
| 9 | *Surgical Procedures, Operative/ | 44,769 |
| 10 | Arthroplasty/ | 10,450 |
| 11 | ((elective or operative or planned) adj3 (surg* or operati* or procedure*)).ti,ab,kf. | 408,771 |
| 12 | (orthop?edic* adj3 (surg* or operati* or procedure*)).ti,ab,kf. | 50,040 |
| 13 | ((hip or knee) adj3 (arthroplast* or surg* or operati* or procedure*)).ti,ab,kf. | 93,808 |
| 14 | arthroplast*.ti,ab,kf. | 100,432 |
| 15 | or/8-14 | 614,687 |
| 16 | exp Empirical Research/ or Interviews as Topic/ or Personal Narratives as Topic/ or Focus Groups/ or exp Narration/ or Nursing Methodology Research/ or Narrative Medicine/ | 208,321 |
| 17 | (Interview or Personal Narrative).pt. | 38,243 |
| 18 | interview*.ti,ab,kf. | 521,019 |
| 19 | qualitative.ti,ab,kf,jw. | 395,842 |
| 20 | (theme* or thematic).ti,ab,kf. | 215,212 |
| 21 | ethnological research.ti,ab,kf. | 8 |
| 22 | ethnograph*.ti,ab,kf. | 15,866 |
| 23 | ethnomedicine.ti,ab,kf. | 1,208 |
| 24 | ethnonursing.ti,ab,kf. | 140 |
| 25 | phenomenol*.ti,ab,kf. | 39,544 |
| 26 | (grounded adj (theor* or study or studies or research or analys?s)).ti,ab,kf. | 17,507 |
| 27 | life stor*.ti,ab,kf. | 1,793 |
| 28 | (emic or etic or hermeneutic* or heuristic* or semiotic*).ti,ab,kf. | 25,139 |
| 29 | (data adj1 saturat$).ti,ab,kf. | 2,797 |
| 30 | participant observ*.ti,ab,kf. | 6,119 |
| 31 | (social construct* or postmodern* or post-structural* or post structural* or poststructural* or post modern* or post-modern*).ti,ab,kf. | 4,862 |
| 32 | (action research or cooperative inquir* or co operative inquir* or co-operative inquir*).ti,ab,kf. | 6,685 |
| 33 | (humanistic or existential or experiential or paradigm*).ti,ab,kf. | 222,327 |
| 34 | (field adj (study or studies or research or work)).ti,ab,kf. | 23,493 |
| 35 | (human science or social science).ti,ab,kf. | 7,986 |
| 36 | biographical method.ti,ab,kf. | 27 |
| 37 | theoretical sampl*.ti,ab,kf. | 1,068 |
| 38 | ((purpos* adj4 sampl*) or (focus adj group*)).ti,ab,kf. | 103,014 |
| 39 | (open-ended or narrative* or textual or texts or semi-structured).ti,ab,kf. | 239,367 |
| 40 | (life world* or life-world* or conversation analys?s or personal experience* or theoretical saturation).ti,ab,kf. | 20,032 |
| 41 | ((lived or life) adj experience*).ti,ab,kf. | 26,145 |
| 42 | cluster sampl*.ti,ab,kf. | 10,916 |
| 43 | observational method*.ti,ab,kf. | 1,124 |
| 44 | content analysis.ti,ab,kf. | 53,730 |
| 45 | (constant adj (comparative or comparison)).ti,ab,kf. | 6,713 |
| 46 | ((discourse* or discurs*) adj3 analys?s).ti,ab,kf. | 3,863 |
| 47 | (heidegger* or colaizzi* or spiegelberg* or merleau* or husserl* or foucault* or ricoeur or glaser*).ti,ab,kf. | 5,657 |
| 48 | (van adj manen*).ti,ab,kf. | 635 |
| 49 | (van adj kaam*).ti,ab,kf. | 49 |
| 50 | (corbin* adj2 strauss*).ti,ab,kf. | 519 |
| 51 | or/16-50 | 1,366,607 |
| 52 | 7 and 15 and 51 | 2,299 |
| 53 | limit 52 to (english language and yr="2009 -Current") | 1,695 |

**Database:**
Embase <1974 to 2025 June 06>

| **#** | **Query** | **Results from 9 Jun 2025** |
| --- | --- | --- |
| 1 | hospital admission/ | 322,144 |
| 2 | time to treatment/ | 32,066 |
| 3 | time factor/ | 51,350 |
| 4 | (wait* adj3 (list* or time*)).ti,ab,kf. | 64,939 |
| 5 | ((delay* or time* or wait*) adj3 (surg* or operati* or procedure* or factor* or treatment*)).ti,ab,kf. | 556,196 |
| 6 | preparation list*.ti,ab,kf. | 13 |
| 7 | or/1-6 | 972,072 |
| 8 | *surgery/ or elective surgery/ or orthopedic surgery/ | 197,820 |
| 9 | hip arthroplasty/ or hip replacement/ | 30,039 |
| 10 | knee arthroplasty/ or knee replacement/ | 29,022 |
| 11 | arthroplasty/ | 23,668 |
| 12 | ((elective or operative or planned) adj3 (surg* or operati* or procedure*)).ti,ab,kf. | 631,566 |
| 13 | (orthop?edic* adj3 (surg* or operati* or procedure*)).ti,ab,kf. | 66,064 |
| 14 | ((hip or knee) adj3 (arthroplasty or surg* or operati* or procedure*)).ti,ab,kf. | 113,262 |
| 15 | arthroplast*.ti,ab,kf. | 120,129 |
| 16 | or/8-15 | 951,448 |
| 17 | exp qualitative research/ or empirical research/ or exp interview/ or narrative/ or verbal communication/ or nursing methodology research/ or narrative medicine/ or content analysis/ or discourse analysis/ or thematic analysis/ or personal experience/ | 673,789 |
| 18 | interview*.ti,ab,kf,dq. | 669,556 |
| 19 | qualitative.ti,ab,kf,jx,dq. | 487,938 |
| 20 | (theme* or thematic).ti,ab,kf,dq. | 263,327 |
| 21 | ethnological research.ti,ab,kf,dq. | 10 |
| 22 | ethnograph*.ti,ab,kf,dq. | 17,324 |
| 23 | ethnomedicine.ti,ab,kf,dq. | 1,899 |
| 24 | ethnonursing.ti,ab,kf,dq. | 143 |
| 25 | phenomenol*.ti,ab,kf,dq. | 43,937 |
| 26 | (grounded adj (theor* or study or studies or research or analys?s)).ti,ab,kf,dq. | 21,479 |
| 27 | life stor*.ti,ab,kf,dq. | 2,246 |
| 28 | (emic or etic or hermeneutic* or heuristic* or semiotic*).ti,ab,kf,dq. | 27,309 |
| 29 | (data adj1 saturat$).ti,ab,kf,dq. | 3,683 |
| 30 | participant observ*.ti,ab,kf,dq. | 6,873 |
| 31 | (social construct* or postmodern* or post-structural* or post structural* or poststructural* or post modern* or post-modern*).ti,ab,kf,dq. | 5,853 |
| 32 | (action research or cooperative inquir* or co operative inquir* or co-operative inquir*).ti,ab,kf,dq. | 8,096 |
| 33 | (humanistic or existential or experiential or paradigm*).ti,ab,kf,dq. | 277,604 |
| 34 | (field adj (study or studies or research or work)).ti,ab,kf,dq. | 26,392 |
| 35 | (human science or social science).ti,ab,kf,dq. | 9,644 |
| 36 | biographical method.ti,ab,kf,dq. | 33 |
| 37 | theoretical sampl*.ti,ab,kf,dq. | 1,215 |
| 38 | ((purpos* adj4 sampl*) or (focus adj group*)).ti,ab,kf,dq. | 132,260 |
| 39 | (open-ended or narrative* or textual or texts or semi-structured).ti,ab,kf,dq. | 287,372 |
| 40 | (life world* or life-world* or conversation analys?s or personal experience* or theoretical saturation).ti,ab,kf,dq. | 25,341 |
| 41 | ((lived or life) adj experience*).ti,ab,kf,dq. | 33,085 |
| 42 | cluster sampl*.ti,ab,kf,dq. | 13,345 |
| 43 | observational method*.ti,ab,kf,dq. | 1,507 |
| 44 | content analysis.ti,ab,kf,dq. | 59,931 |
| 45 | (constant adj (comparative or comparison)).ti,ab,kf,dq. | 8,407 |
| 46 | ((discourse* or discurs*) adj3 analys?s).ti,ab,kf,dq. | 4,324 |
| 47 | (heidegger* or colaizzi* or spiegelberg* or merleau* or husserl* or foucault* or ricoeur or glaser*).ti,ab,kf,dq. | 6,169 |
| 48 | (van adj manen*).ti,ab,kf,dq. | 709 |
| 49 | (van adj kaam*).ti,ab,kf,dq. | 54 |
| 50 | (corbin* adj2 strauss*).ti,ab,kf,dq. | 569 |
| 51 | or/17-50 | 1,803,737 |
| 52 | 7 and 16 and 51 | 4,543 |
| 53 | limit 52 to (english language and yr="2009 -Current") | 3,977 |

**Database:**
Ovid Emcare <1995 to 2025 Week 22>

| **#** | **Query** | **Results from 9 Jun 2025** |
| --- | --- | --- |
| 1 | hospital admission/ | 119,360 |
| 2 | time to treatment/ | 10,081 |
| 3 | time factor/ | 3,852 |
| 4 | (wait* adj3 (list* or time*)).ti,ab,kf. | 18,788 |
| 5 | ((delay* or time* or wait*) adj3 (surg* or operati* or procedure* or factor* or treatment*)).ti,ab,kf. | 119,611 |
| 6 | preparation list*.ti,ab,kf. | 6 |
| 7 | or/1-6 | 256,945 |
| 8 | *surgery/ or elective surgery/ or orthopedic surgery/ | 69,943 |
| 9 | hip arthroplasty/ or hip replacement/ | 13,480 |
| 10 | knee arthroplasty/ or knee replacement/ | 12,260 |
| 11 | arthroplasty/ | 12,943 |
| 12 | ((elective or operative or planned) adj3 (surg* or operati* or procedure*)).ti,ab,kf. | 126,300 |
| 13 | (orthop?edic* adj3 (surg* or operati* or procedure*)).ti,ab,kf. | 27,408 |
| 14 | ((hip or knee) adj3 (arthroplasty or surg* or operati* or procedure*)).ti,ab,kf. | 60,779 |
| 15 | arthroplast*.ti,ab,kf. | 65,035 |
| 16 | or/8-15 | 264,753 |
| 17 | exp qualitative research/ or empirical research/ or exp interview/ or narrative/ or verbal communication/ or nursing methodology research/ or narrative medicine/ or content analysis/ or discourse analysis/ or thematic analysis/ or personal experience/ | 470,118 |
| 18 | interview*.ti,ab,kf,dq. | 368,136 |
| 19 | qualitative.ti,ab,kf,jx,dq. | 264,234 |
| 20 | (theme* or thematic).ti,ab,kf,dq. | 167,212 |
| 21 | ethnological research.ti,ab,kf,dq. | 3 |
| 22 | ethnograph*.ti,ab,kf,dq. | 15,620 |
| 23 | ethnomedicine.ti,ab,kf,dq. | 566 |
| 24 | ethnonursing.ti,ab,kf,dq. | 123 |
| 25 | phenomenol*.ti,ab,kf,dq. | 26,189 |
| 26 | (grounded adj (theor* or study or studies or research or analys?s)).ti,ab,kf,dq. | 16,383 |
| 27 | life stor*.ti,ab,kf,dq. | 1,783 |
| 28 | (emic or etic or hermeneutic* or heuristic* or semiotic*).ti,ab,kf,dq. | 13,828 |
| 29 | (data adj1 saturat$).ti,ab,kf,dq. | 2,089 |
| 30 | participant observ*.ti,ab,kf,dq. | 6,114 |
| 31 | (social construct* or postmodern* or post-structural* or post structural* or poststructural* or post modern* or post-modern*).ti,ab,kf,dq. | 5,810 |
| 32 | (action research or cooperative inquir* or co operative inquir* or co-operative inquir*).ti,ab,kf,dq. | 6,336 |
| 33 | (humanistic or existential or experiential or paradigm*).ti,ab,kf,dq. | 80,866 |
| 34 | (field adj (study or studies or research or work)).ti,ab,kf,dq. | 6,906 |
| 35 | (human science or social science).ti,ab,kf,dq. | 5,211 |
| 36 | biographical method.ti,ab,kf,dq. | 24 |
| 37 | theoretical sampl*.ti,ab,kf,dq. | 960 |
| 38 | ((purpos* adj4 sampl*) or (focus adj group*)).ti,ab,kf,dq. | 84,893 |
| 39 | (open-ended or narrative* or textual or texts or semi-structured).ti,ab,kf,dq. | 173,765 |
| 40 | (life world* or life-world* or conversation analys?s or personal experience* or theoretical saturation).ti,ab,kf,dq. | 10,317 |
| 41 | ((lived or life) adj experience*).ti,ab,kf,dq. | 21,800 |
| 42 | cluster sampl*.ti,ab,kf,dq. | 6,019 |
| 43 | observational method*.ti,ab,kf,dq. | 850 |
| 44 | content analysis.ti,ab,kf,dq. | 45,745 |
| 45 | (constant adj (comparative or comparison)).ti,ab,kf,dq. | 5,831 |
| 46 | ((discourse* or discurs*) adj3 analys?s).ti,ab,kf,dq. | 4,823 |
| 47 | (heidegger* or colaizzi* or spiegelberg* or merleau* or husserl* or foucault* or ricoeur or glaser*).ti,ab,kf,dq. | 5,072 |
| 48 | (van adj manen*).ti,ab,kf,dq. | 656 |
| 49 | (van adj kaam*).ti,ab,kf,dq. | 43 |
| 50 | (corbin* adj2 strauss*).ti,ab,kf,dq. | 529 |
| 51 | or/17-50 | 824,770 |
| 52 | 7 and 16 and 51 | 1,202 |
| 53 | limit 52 to (english language and yr="2009 -Current") | 1,001 |

CINAHL Complete (Ebsco)

| **Search ID#** | **Search Terms** | **Search Options** | **Last Run Via** | **Results** |
| --- | --- | --- | --- | --- |
| S20 | S6 AND S17 AND S18 | Limiters - Publication Date: 20090101-20250531; English Language Search modes - Proximity | Interface - EBSCOhost Research Databases Search Screen - Advanced Search Database - CINAHL Complete | 472 |
| S19 | S6 AND S17 AND S18 | Search modes - Proximity | Interface - EBSCOhost Research Databases Search Screen - Advanced Search Database - CINAHL Complete | 620 |
| S18 | MH Qualitative Studies OR MH Grounded theory OR MH Narratives OR MH Interviews+ OR MH Audiorecording OR MH Focus Groups OR MH Research, Nursing OR MH Discourse Analysis OR MH Content Analysis OR MH Ethnographic Research OR MH Ethnonursing Research OR MH Constant Comparative Method OR MH Qualitative Validity+ OR MH Purposive Sample OR MH Observational Methods+ OR MH Field Studies OR MH Theoretical Sample OR MH Phenomenology OR MH Phenomenological Research OR MH Life Experiences+ OR MH Cluster Sample+ OR TI qualitative OR AB qualitative OR TI interview* OR AB interview* OR TI (theme* or thematic) OR AB (theme* or thematic) OR TI ("ethnological research") OR AB ("ethnological research") OR TI ethnonursing OR AB ethnonursing OR TI ethnograph* OR AB ethnograph* OR TI phenomenol* OR AB phenomenol* OR TI "focus group*" OR AB "focus group*" OR TI (grounded N1 (theor* OR analys?s OR research OR studies OR study)) OR AB (grounded N1 (theor* OR analys?s OR research OR studies OR study)) OR TI ("life stor*") OR AB ("life stor*") OR TI (emic OR etic OR hermeneutic* OR heuristic* OR semiotic) OR AB (emic OR etic OR hermeneutic* OR heuristic* OR semiotic) OR TI (data N1 saturat*) OR AB (data N1 saturat*) OR TI ("participant observ*") OR AB ("participant observ*") OR TI ("social construct*" OR postmodern* OR "post-structural*" OR poststructural* OR "post-modern*" OR feminis*) OR AB ("social construct*" OR postmodern* OR "post-structural*" OR poststructural* OR "post-modern*" OR feminis*) OR TI ("action research" OR "cooperative inquir*" OR "co-operative inquir*") OR AB ("action research" OR "cooperative inquir*" OR "co-operative inquir*") OR TI (humanistic OR existential OR experiential OR paradigm*) OR AB (humanistic OR existential OR experiential OR paradigm*) OR TI (field N1 (research OR study OR studies)) OR AB (field N1 (research OR study OR studies)) OR TI "human science" OR AB "human science" OR TI "biographical method" OR AB "biographical method" OR TI ("theoretical sampl*") OR AB ("theoretical sampl*") OR TI ("purpos* N4 sampl*") OR AB ("purpos* N4 sampl*") OR TI ("open-ended" OR narrative* OR textual OR texts OR "semi-structured") OR AB ("open-ended" OR narrative* OR textual OR texts OR "semi-structured") OR TI ("life world" OR "life-world" OR "conversation analys?s" OR "personal experience*" OR "theoretical saturation") OR AB ("life world" OR "life-world" OR "conversation analys?s" OR "personal experience*" OR "theoretical saturation") OR TI ((life OR lived) N1 experience*) OR AB ((life OR lived) N1 experience*) OR TI ("cluster sampl*") OR AB ("cluster sampl*") OR TI ("observational method*") OR AB ("observational method*") OR TI ("content analysis") OR AB ("content analysis") OR TI ((discurs* OR discourse*) N3 analys?s) OR AB ((discurs* OR discourse*) N3 analys?s) OR TI (constant N1 (comparison OR comparative)) OR AB (constant N1 (comparison OR comparative)) OR TI ("narrative analys?s") OR AB ("narrative analys?s") OR TI (heidegger* OR colaizzi* OR spiegelberg* OR merleau* OR husserl* OR foucault* OR ricoeur OR glaser*) OR AB (heidegger* OR colaizzi* OR spiegelberg* OR merleau* OR husserl* OR foucault* OR ricoeur OR glaser*) OR TI (van N1 manen*) OR AB (van N1 manen*) OR TI (van N1 kaam*) OR AB (van N1 kaam*) OR TI (Corbin* N2 strauss*) OR AB (Corbin* N2 strauss*) | Search modes - Proximity | Interface - EBSCOhost Research Databases Search Screen - Advanced Search Database - CINAHL Complete | 803,151 |
| S17 | S7 OR S8 OR S9 OR S10 OR S11 OR S12 OR S13 OR S14 OR S15 OR S16 | Search modes - Proximity | Interface - EBSCOhost Research Databases Search Screen - Advanced Search Database - CINAHL Complete | 127,607 |
| S16 | TI arthroplast* OR AB arthroplast* | Search modes - Proximity | Interface - EBSCOhost Research Databases Search Screen - Advanced Search Database - CINAHL Complete | 42,377 |
| S15 | TI ( (hip or knee) N2 (arthroplast* or surg* or operati* or procedure*) ) OR AB ( (hip or knee) N2 (arthroplast* or surg* or operati* or procedure*) ) | Search modes - Proximity | Interface - EBSCOhost Research Databases Search Screen - Advanced Search Database - CINAHL Complete | 41,321 |
| S14 | TI ( orthop#edic* N2 (surg* or operati* or procedure*) ) OR AB ( orthop#edic* N2 (surg* or operati* or procedure*) ) | Search modes - Proximity | Interface - EBSCOhost Research Databases Search Screen - Advanced Search Database - CINAHL Complete | 16,720 |
| S13 | TI ( (elective or operative or planned) N2 (surg* or operati* or procedure*) ) OR AB ( (elective or operative or planned) N2 (surg* or operati* or procedure*) ) | Search modes - Proximity | Interface - EBSCOhost Research Databases Search Screen - Advanced Search Database - CINAHL Complete | 17,961 |
| S12 | (MH "Arthroplasty") | Search modes - Proximity | Interface - EBSCOhost Research Databases Search Screen - Advanced Search Database - CINAHL Complete | 3,906 |
| S11 | (MM "Surgery, Operative") | Search modes - Proximity | Interface - EBSCOhost Research Databases Search Screen - Advanced Search Database - CINAHL Complete | 16,087 |
| S10 | (MH "Knee Surgery") OR (MH "Arthroplasty, Replacement, Knee") | Search modes - Proximity | Interface - EBSCOhost Research Databases Search Screen - Advanced Search Database - CINAHL Complete | 22,236 |
| S9 | (MH "Hip Surgery+") | Search modes - Proximity | Interface - EBSCOhost Research Databases Search Screen - Advanced Search Database - CINAHL Complete | 23,483 |
| S8 | (MH "Orthopedic Surgery") | Search modes - Proximity | Interface - EBSCOhost Research Databases Search Screen - Advanced Search Database - CINAHL Complete | 20,172 |
| S7 | (MH "Surgery, Elective") | Search modes - Proximity | Interface - EBSCOhost Research Databases Search Screen - Advanced Search Database - CINAHL Complete | 7,236 |
| S6 | S1 OR S2 OR S3 OR S4 OR S5 | Search modes - Proximity | Interface - EBSCOhost Research Databases Search Screen - Advanced Search Database - CINAHL Complete | 265,566 |
| S5 | TI "preparation list*" OR AB "preparation list*" | Search modes - Proximity | Interface - EBSCOhost Research Databases Search Screen - Advanced Search Database - CINAHL Complete | 3 |
| S4 | TI ( (delay* or time* or wait*) N2 (surg* or operati* or procedure* or factor* or treatment*) ) OR AB ( (delay* or time* or wait*) N2 (surg* or operati* or procedure* or factor* or treatment*) ) | Search modes - Proximity | Interface - EBSCOhost Research Databases Search Screen - Advanced Search Database - CINAHL Complete | 70,465 |
| S3 | TI ( wait* N2 (list* or time*) ) OR AB ( wait* N2 (list* or time*) ) | Search modes - Proximity | Interface - EBSCOhost Research Databases Search Screen - Advanced Search Database - CINAHL Complete | 13,622 |
| S2 | (MH "Time Factors") | Search modes - Proximity | Interface - EBSCOhost Research Databases Search Screen - Advanced Search Database - CINAHL Complete | 189,677 |
| S1 | (MH "Waiting Lists") | Search modes - Proximity | Interface - EBSCOhost Research Databases Search Screen - Advanced Search Database - CINAHL Complete | 7,446 |
